# Block Copolymers in 3D/4D Printing: Advances and Applications as Biomaterials

**DOI:** 10.3390/polym15020322

**Published:** 2023-01-08

**Authors:** Nikolaos Politakos

**Affiliations:** POLYMAT, Applied Chemistry Department, Faculty of Chemistry, University of the Basque Country, UPV/EHU, Paseo Manuel de Lardizabal 3, 20018 Donostia-San Sebastián, Spain; nikolaos.politakos@ehu.eus

**Keywords:** block copolymers, 3D printing, 4D printing, biomaterials, scaffolds, tissue engineering

## Abstract

3D printing is a manufacturing technique in constant evolution. Day by day, new materials and methods are discovered, making 3D printing continually develop. 3D printers are also evolving, giving us objects with better resolution, faster, and in mass production. One of the areas in 3D printing that has excellent potential is 4D printing. It is a technique involving materials that can react to an environmental stimulus (pH, heat, magnetism, humidity, electricity, and light), causing an alteration in their physical or chemical state and performing another function. Lately, 3D/4D printing has been increasingly used for fabricating materials aiming at drug delivery, scaffolds, bioinks, tissue engineering (soft and hard), synthetic organs, and even printed cells. The majority of the materials used in 3D printing are polymeric. These materials can be of natural origin or synthetic ones of different architectures and combinations. The use of block copolymers can combine the exemplary properties of both blocks to have better mechanics, processability, biocompatibility, and possible stimulus behavior via tunable structures. This review has gathered fundamental aspects of 3D/4D printing for biomaterials, and it shows the advances and applications of block copolymers in the field of biomaterials over the last years.

## 1. Introduction

Scientific progress is changing rapidly from day to day, leading to new advances in many different aspects of research. Many areas of investigation are transforming, making the advances important every day. More and more new techniques and advances are imposed in scale-up on an industrial scale. An important method in the manufacturing of materials is considered 3D printing [1]. It is an area of investigation constantly evolving and moving towards, in terms of improving the existing pieces of equipment, using new materials, and preparing objects that are more complex. This evolution in the research in materials used in this technique creates the need to develop novel materials that can be printable and have properties for specific needs and areas. 

3D was introduced as a manufacturing technique in the 80s and immediately gained attention. Now it is becoming one of the leading methods of fabrication, especially for hierarchically complex architectures, which are not always achievable by conventional manufacturing technologies [1]. This technique has proven to be versatile, and this is mainly because of its operational simplicity. 3D printing can be used in many different areas, such as electronics, education, robotics, sensing, the pharmaceutical industry, design, aerospace, automation, and biomedical engineering [1].

Nowadays, 3D printing production can use different materials, such as metals, ceramics, and polymers without the need for molds or machining typical for conventional formative and subtractive fabrication [2]. The cost of a 3D printer can be less than $500, enabling desktop fabrication of 3D objects even at home [2]. Since the 1980s, there has been a considerable jump in scientific and technological impact in manufacturing [2]. 3D printing is the primary reason for this. Apart from the scientific part, the economic impact is also tremendous since, with 3D printing, there was a change in product development due to lower cost and acceleration in manufacturing. The overall market has significant growth rates and surpassed the value of 5 billion USD in 2015 [2].

Nevertheless, interest in 3D printing has increased over the last 25 years, but bioprinting is still young. It has constantly been developing over the last 10 years, as seen from increased patents and publications. 3D printing has great potential to become the major player in manufacturing complex materials for many applications in the following years. This growth is accomplished by constantly researching new materials with exciting properties and use in wide applications [2,3,4,5,6,7].

This review aims to explore block copolymers’ major contribution to 3D printing in recent years by focusing on bio-applications. It is well known that one of the major materials that are currently in use for 3D printing are polymers. Most of the polymers used in 3D printing in bioprinting are homopolymers. Block copolymers constantly evolve through new synthetic approaches and introducing new blocks with stimulating properties. This review aims to manifest the use of block copolymers as a leading evolving player in 3D/4D printing. This review will try to explore the significant contribution of block copolymers in bio-applications with specific advances.

## 2. Aspects on 3D and 4D Printing

**Figure 1:**
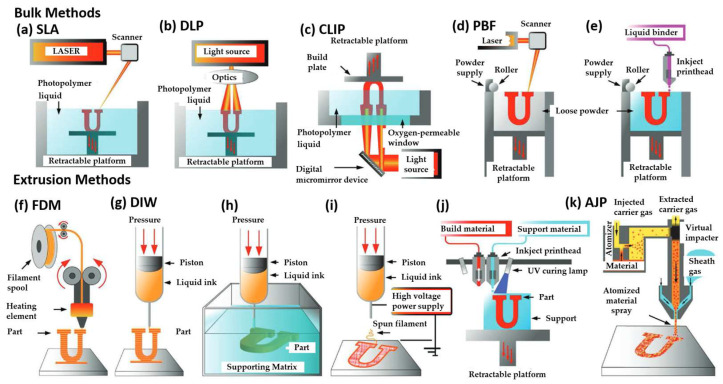
Schematics of bulk and extrusion 3D printing methods. (**a**–**e**) Bulk methods: (**a**) top-down laser stereolithography (SLA), (**b**) top-down digital projection lithography (DLP), (**c**) continuous liquid interface production (CLIP), (**d**) powder bed fusion (PBF), and (**e**) binder jet. (**f**–**k**) Extrusion methods: (**f**) fused deposition modeling (FDM), (**g**) direct ink writing (DIW), (**h**) sacrificial/embedded printing, (**i**) electrospinning, (**j**) direct inkjet printing, and k) aerosol jet printing (AJP). Reprinted with permission from [9].

3D printing is the additive fabrication process in which a material is joined or solidified to form complex geometric structures controlled by a computer. The term was introduced by Emanuel Sachs et al. [8] in 1989 with the invention of binder jetting 3D printing technology. The truth is that the concept has existed since 1986 with the use of stereolithography [9]. 3D printing can be categorized into two general categories in terms of preparation of the structures. The first is considered the “bulk” method, and the second is the “extrusion” method. Bulk methods are based on technologies that can print directly in an extensive reservoir of raw material by selectively curing, sintering, or binding material in a layer-by-layer fashion [9]. Extrusion methods are distinguished by the process of the material that is selectively deposited on a print bed from a separate reservoir. These techniques can be seen in Figure 1. 

Bulk methods are considered Figure 1a top-down laser stereolithography (SLA), Figure 1b top-down digital projection lithography (DLP), Figure 1c continuous liquid interface production (CLIP), Figure 1d powder bed fusion (PBF), and Figure 1e binder jet. On the other hand, extrusion methods are Figure 1f fused deposition modeling (FDM), Figure 1g direct ink writing (DIW), Figure 1h sacrificial/embedded printing, Figure 1i electrospinning, Figure 1j direct inkjet printing and Figure 1k aerosol jet printing (AJP). Table 1 gathered information about each technique as long, including basic characteristics of resolution and speed [9]. As can be seen from Table 1, numerous techniques are being used depending on what needs exist for manufacturing different structures. The choice of the method is based mainly on the type of material that is going to be used and the limitations that it has in terms of processability. According to different techniques, the printing speed and the structure’s final resolution can be affected.

3D printing is a technique where, in order to show optimized results, efficiency and precision are essential. Specific parameters must be taken into consideration. These parameters can be based on three main ones [10]:

1. Geometry related (nozzle size and filament size)
2. Process related (melting temperature, bed temperature, and printing speed)
3. Structural related (layer thickness, infill geometry-density, raster angle-gap)

**Table 1:** 3D printing techniques with its basic characteristics [9].

3D Printing Technique	Material	Resolution Speed	Description
Stereolithography (SLA)	Photocurable resin	50–200 μm [11] 1000 mL/h [12]	Use of ultraviolet (UV) laser to polymerize a photocurable resin layer by layer
Digital projection lithography (DLP)	Photocurable resin	1 μm [13] ≈50 mm/h [14,15,16,17]	Use of UV light to selectively polymerize a liquid resin with a spatial light modulating element
Continuous liquid interface production (CLIP)	Photocurable resin	10–100 μm [18] 500 mm/h [19]	Use of a similar projection method to DLP, with the addition of an oxygen permeable window
Two-photon polymerization (2PP)	Photocurable resin	100 nm [11] 80 nm/s–2 cm/s [13]	Use near-infrared femtosecond laser pulses to polymerize a nanoscale voxel at the focal point of the laser
Powder bed fusion (PBF)	Polymer Metal Ceramic	20–100 μm [14,15,16,17,18,19,20] 1000 mL/h [15,16,17,18,19,20,21]	Uses a high-power photon or electron source to fuse the selectively powder layer by layer, while fresh powder is spread onto the previously bonded layer
Binder jet	Polymer Metal Ceramic compatible liquid binder	50–400 μm [22] 25 mm/h [23]	Jets tiny droplets of binder onto a polymer, metal, or ceramic powder using an inkjet printhead
Fused deposition modeling (FDM)	Thermoplastic filament	100 μm [12] 100 mL/h [12]	Uses rollers to push thermoplastic filament through a heated metal nozzle
Direct ink writing (DIW)	Viscoelastic ink Shear thinning fluid	1–250 μm [11] 100 mL/h [12]	Extrudes liquid ink through a nozzle or needle
Sacrificial/embedded printing	Ink compatible with DIW process. Support: shear thinning fluid, or high viscosity reservoir and low viscosity filler combination	1–250 μm [11] 1300 mL/h [24]	The nozzle of an ink dispensing system is inserted into a matrix of soft material. The supporting structure allows the ink to be 3D printed by tracing a 3D trajectory
Electro-hydrodynamic printing (EHD)	Polymer-based solution	100 nm–20 µm [25] 20–1500 mL/h [26]	Use a voltage between the nozzle and substrate to eject fluid from the nozzle
Direct inkjet printing	Low-viscosity fluid	240 nm–5 µm [12] 500 mL/h [27]	Deposition of droplets by means of a valve inside the printhead, formed by electrostatic, thermal, or piezoelectric plates
Aerosol jet printing (AJP)	Metal inks biological inks adhesives polymers	10 µm [28] 1200 mL/h [29]	Uses aerodynamic focusing to guide a narrow spray of atomized fluid onto a substrate

3D printing manufactures objects with properties and shapes that do not change during their entire product life. 4D printing changes the aspect mentioned above by fabricating “smart” structures that can alter their shape after external stimulus, such as light, shear, water, touch, pH, electromagnetic field, or temperature [30,31,32]. Easily it can be hypothesized that this type of printing can open new roads to the fabrication of materials with use in applications with high demands. 4D printing can create dynamic structures that can easily be used as biological structures, hydrogels, or stimuli-responsive shapes [2].

One of the first 4D printing was adopted by Skylar Tibbits in fabricating materials that expand at different rates [33]. These materials stretch and fold to form different shapes when activated by the stimulus [33]. Currently, three different approaches can be recognized:

1. Smart materials that change their shape upon stimuli.
2. 3D printing materials that can act as supporting structures for growth of organic cells.
3. Self-assembly of micro-sized smart particles that, upon stimulus change their pattern.

4D printing has differences in the technologies used for printing, mainly in using smart polymers that are used with traditional 3D printing techniques such as stereolithography, fused deposition modeling, inkjet printing, and others [34]. With slight modifications to the traditional 3D printing technologies, an adaptative 4D technology can be used [35]. More specifically, the FDM technique was modified by adding an air circulation system for 4D printing (cooling down the system below its glass transition temperature). Inkjet printing is also used in manufacturing cells since it is highly biocompatible [35]. DIW can also be used with specific bio-inks to prepare biomedical scaffolds and tissue engineering. SLA is also a technique that is used for the preparation of networks. Modification of SLA with UV-LED parts and projection micro stereolithography (PSL) can be used for structures with shape memory ability that can be used in drug delivery systems [33].

One of the most critical steps toward the evolution of 3D printing is the introduction of functional polymers. These polymers have a specific structure and properties that can be altered and perform different actions (physical, chemical, or biological) based on external stimuli (pH, light, temperature, mechanical loading, or voltage) [36]. The changes happening to these materials are through thermodynamics (change in hydrophobicity), protonation or deprotonation, polarization, rearrangement, and cleavage of bonds [36]. These small changes can lead to macroscopic changes in these materials, is swelling or aggregation, changing of color, or shrinking [37,38,39]. Specific reference should be made to self-healing polymers that can recover from partial destruction, which can be attributed to the dynamic nature of their bonds and the reversibility of the damage through reformation [40].

3D printing is becoming a versatile technique for manufacturing advanced functional materials with specific properties for many applications. Last few years, this technique has become more approachable to more people and industry. In the following years, 3D printing will have a significant role in energy, food production, medicine, and engineering. In particular, in the medicinal field, it can bring revolution to orthopedics, implants, tissue engineering, scaffolds, drug delivery, and regenerative medicine [1].

## 3. Bioprinting

Biomaterials are an emerging field. These materials are constantly evolving and developing, and they have a great share in the global market with an increased growth rate [41]. 3D printing is a technique that can be used in the field of biomaterials, providing top-of-the-edge constructions for applications with specific properties. In the last years, 3D printing has increased in popularity as a bioprinting technique because has great potential. Bioprinting can help scientists to fabricate tissues, organs, biological systems, drug delivery systems, and other in vitro systems that mimic their natural counterparts [42]. Bioprinting is defined by Groll et al. [43] as the process of using computer-aided transfer processes to pattern and assembles biologically relevant materials, including molecules, cells, tissues, and biodegradable biomaterials based on prescribed 2D or 3D originations resulting in the formation of the engineered biofunctional construct [43].

In bioprinting, a plethora of materials can be used to achieve the goal, such as cells, drugs, genes, growth factors, and of course, polymers (synthetic or natural). Bioprinting is an interdisciplinary field with a combination of knowledge from chemistry, biology, medicine, computer science, and materials science. In order to achieve the goal, many different parameters must be kept in mind. The bioprinter is the most important part of constructing the biomaterial apart from the material itself. This makes it clear that parameters such as build speed, user-friendliness, full automation capability, ease of sterilization, affordability, versatility, and compactness are important [42]. Conventional manufacturing techniques are not at the top of preparing biomaterials with complete geometry control (pore size, porous network, and porosity). Bioprinting as a fabrication method seems to have the potential to produce complex living and nonliving biological products from cells, molecules, extracellular matrices, and biomaterials [41].

### 3.1. Techniques in Bioprinting

In the field of bioprinting, four main strategies exist for manufacturing objects via 3D. The fabrication technologies include lithography-based [44,45,46], drop-on-demand [42,47,48], laser-induced forward transfer [49,50], and extrusion-based [51]. In Table 2, these techniques and their advantages and disadvantages can be found. Of these strategies, the most commonly used is extrusion-based, mainly because of its versatility, affordability, and capability to construct complex structures [52]. Tissues, living cells, organ modules, and organ-on-a-chip are some of the examples that can be 3D printed. The advantages of extrusion n-based are the commercial affordability, capability for printing high cell density bioink, and creating 3D complex structures of materials and multiple cell types [53,54,55]. For bioprinting, the techniques are less than the standard 3D printing. The most crucial parameter that someone needs to consider is the viability of the cells and, of course, the resolution. Then, some disadvantages are parameters related to the cost and processability, such as poor quality, limitation to specific materials, printing speed, and particular viscosities. Nevertheless, from the abovementioned techniques, it can be seen that now it can print blood vessels, bone, and cartilage, and for other applications, a specific method is used.

The different techniques used in biofabrication have specific applications based on their limitations, advantages, and disadvantages. 3D printing can be used in scaffolds, hydrogels, tissue engineering, and cell growth. Extrusion-based methods are currently used in pharmaceuticals, scaffolds, bone tissue engineering, and cardiovascular medical devices. Finally, other indirect methods (stereolithography or selective laser sintering) can be used in pharmaceutical, biomedical manufacturing, bone tissue engineering, and drug delivery [56].

**Table 2:** Comparison of four types of bioprinting methods. Combining information from [52].

Printing Method	Advantages	Disadvantages	Applications	Refs.
Drop on demand	Low cost Fast print speed High resolution High cell viability (>85%)	Low cell density (<10^6^ cells/mL) Poor quality of vertical structures Bioink with specific range of viscosity	Blood vessel Bone Cartilage Neuron	[57,58,59,60]
Lithography based	Low cost High cell viability (>85%) High resolution Good vertical structure fidelity Fast printing speed	Limited to photopolymerization Medium on cell density (<10^8^ cells/mL)	Blood vessel Cartilage Bone	[61,62,63,64,65,66,67,68,69,70]
Laser assisted	High cell viability (>95%) High resolution Fair vertical structure fidelity	Expensive Medium printing speed Bioink with specific range of viscosity Medium cell density (<10^8^ cells/mL)	Blood vessel Bone Skin Adipose	[71,72,73,74]
Extrusion based	Moderate resolution Cell-laden bioink Good vertical structure fidelity Supports high viscosity bioink	Fairly expensive slow printing speed low cell viability (40–80%)	Blood vessel Bone Cartilage Neuron Muscle Tumor	[61,62,63,64,65,66,67,68,69]

### 3.2. Criteria and Limitations

3D printing has its limitations in terms of materials and technical parameters used during the formulation of the objects for applications in bio. It is important to notice that specific things must be considered since it has to deal with sensitive biological materials. The most important is to control temperature (range between 5–50 °C), pH (range between 6.5–7.8), irradiation, organic solvents (avoided), and pressure (high pressure can destroy cells) to ensure the viability of the structures prepared [75].

**Figure 2:**
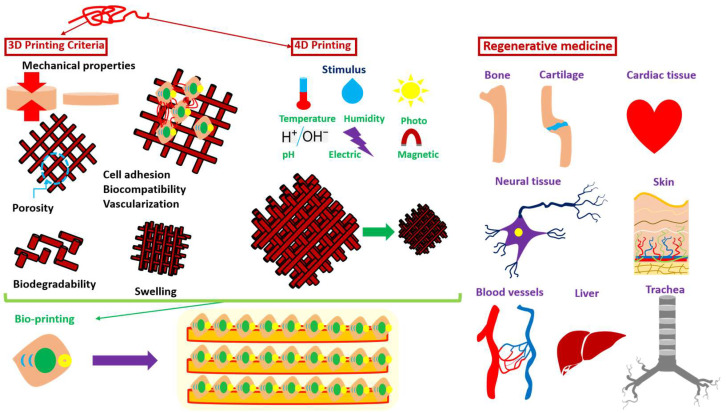
Schematic illustration of the criteria that should be considered for tissue engineering, differences of 3D Vs 4D (bio)-printing and main organs/tissues in regenerative medicine currently under investigation.

In the case of tissue engineering, for example, many criteria should be considered, especially for the case of the hydrogels used (Figure 2). These criteria are [76]:

1. Mechanical properties: tailored to meet specific end-user requirements.
2. Biodegradability/biosorbability: ideally bioresorbability and tunable degradation upon formation of functional tissues.
3. Porosity: porosity or hierarchical transport properties is vital for efficient nutrient and metabolic waste transport and optimal cell migration.
4. Swelling: crucial function in materials diffusion and transport within and through the hydrogel (cell stability and molecule release for drug delivery).
5. Biocompatibility: integrated into the biological system without harming or rejected (minimal or no immune reactions).
6. Cell adhesion: display adhesion property for cell binding.
7. Vascularization: capillary network responsible for nutrients transport to the cells.
8. Bioactivity: trigger/facilitate a biological response within a living system (tissue interactivity and binding ability, excellent osteoconductivity and osteoinductivity, and cell differentiation, attachment, and ingrowth).

### 3.3. Bioprinting in Regenerative Medicine

Many polymeric materials exist for 3D printing organs or tissues and are under investigation. The research in organs/tissues is concentrated in bone, cartilage, heart valve, cardiac tissue, neural tissue, blood vessels, trachea, liver, and skin in regenerative medicine is vast (Figure 2). Materials that are currently used are based on natural polymers (agarose, alginate, chitosan, collagen, and gelatin) or synthetic ones (homopolymers and copolymers) [41]. For the case of the synthetic polymers, it can be found that the majority of them are based on Polycaprolactone (PCL), Polyurethane (PU), Polyethylene Glycol (PEG), Polylactic-co-glycolic Acid (PLGA), Pluronic Acid (or Poloxamer), and Polydimethylsiloxane (PDMS) [77]. More specifically, in the above mentioned areas, research is concentrated on some polymers (synthetic or natural) with specific properties.

#### 3.3.1. Bone

Bone is one of the most studied areas in 3D printing. Synthetic biodegradable polymers that are currently under investigation are based on poly(caprolactone) (PCL), poly(glycolic acid) (PGA), poly(lactic acid) (PLA), and their copolymers. These materials can undergo hydrolysis, and they produce non-toxic materials. Scaffolds from these materials can be prepared with similar properties to the natural bone [78].

#### 3.3.2. Cartilage

As in the case of bone, this is one of the most studied areas. Bioink can be used in order to mimic shapes from simple to more complex. Works are using thermo-sensitive methacrylate pHPMA-lac-PEG triblock copolymer with chondrocytes showing nice mechanical properties, stability in the long term, and processability via printing [79]. In another work, PCL and alginate hydrogel were used with incorporated embryonic chick cartilage cells proving increased cell viability, proliferation, and differentiation [80]. Other materials that can be used are Poly(ethylene glycol) terephthalate/poly(butylene terephthalate) (PEGT/PBT) block copolymer (grid-like structured scaffold) or an inorganic-organic hybrid of silica poly(tetrahydrofuran)/PCL for fabricating scaffolds for articular cartilage regeneration [78].

#### 3.3.3. Cardiac Tissue

In this case, synthetic polymers that can be used are based on alginate with human cardiac-derived cells, increasing cell viability and retention of the cardiac lineage. Moreover, PEUU was also used in in vivo studies showing increased vessel formation and integration of cells [41].

#### 3.3.4. Heart Valve

Materials used in 3D printing involving PLA, fibrin, collagen, and PGA. In this case, mechanical properties are essential. Methacrylated hyaluronic acid hydrogels have been used, showing cell viability and glycosaminoglycan matrix formation. Bioprinted alginate/gelatin hydrogels were also prepared with high viability and sound biomechanics [41,78].

#### 3.3.5. Neural Tissue

Astrocytes and neurons from embryonic rats were bioprinted in a 3D collagen gel acting as a scaffold. Finally, an artificial neural tissue with murine neural stem cells, collagen hydrogels, and fibrin gels was fabricated, showing high cell viability [41].

#### 3.3.6. Blood Vessels

This area is highly important since there is a high demand for artificial blood vessels. Ideally, an artificial blood vessel should be anti-thrombogenic, durable, biocompatible, and have comparable structural properties to the native ones. 3D bioprinted collagen was used with fugitive ink composed of gelatin with endothelial cells, showing a high potential for practical use. Another natural polymer is based on fibrin by polymerizing fibrinogen and thrombin in creating scaffolds. Finally, a 3D printed aorta based on poly(propylene fumarate) was fabricated using digital light stereolithography, with bioactive properties in vivo and comparable mechanical strength [78].

#### 3.3.7. Trachea

Here, a work based on PCL powder with hydroxyapatite was used for preparing scaffolds. MSC-seeded fibrin coated the scaffold to improve bioactivity; in vivo studies showed reconstruction of the trachea and mechanically stable structures. Other works involve the methacrylated silk fibroin crosslinked via UV. This cell-loaded hydrogel scaffold showed excellent results in vitro [78].

#### 3.3.8. Liver

The liver is one of the most fundamental organs in the human body with essential functions. A work is related with gelatin hydrogel with hepatocyte as an extracellular matrix (ECM), crosslinked with glutaraldehyde. In vitro experiments showed the survival of the cells over two months. A chitosan-gelatin scaffold was prepared to mimic the natural liver in architecture. This hybrid scaffold was highly porous with a well-organized structure [78].

#### 3.3.9. Skin

The skin is an area in which many applications of 3D printing can be found, especially in the case of skin burns. Collagen is one of the obvious candidates for use since it is a big part of the natural skin. With bioprinting using fibroblasts and keratinocytes, fully functional skin was prepared. In vivo experiments revealed the epidermis’ formation and blood vessels’ presence in the wound area [41].

As 3D printing techniques evolve and more complex structures are prepared, this opens the road for printing different organs and more complex systems of the human body. The printing of bone and skin can move towards printing blood vessels and neurons, as already seen from the numerous kinds of research in biomaterials.

### 3.4. Bioinks

Bioinks are one of the most important ways of fabricating structures in bioprinting, especially with stereolithography. The bioinks used in printing must have certain aspects, such as biocompatibility, printability, and robustness [81]. The main types of bioinks are microcarriers, cell aggregates, decellularized components, and most importantly, hydrogels [42]. Hydrogels are considered one of the best choices as bioinks can easily be loaded with cells, can be crosslinked, have a high swelling degree, printability, and biocompatibility [81]. Hydrogels can be widely categorized into natural and synthetic ones. Natural ones that are currently used or investigated are based mainly on polysaccharides (agarose, alginate, and chitosan) and components of ECM (fibronectin, laminin, collagen, and gelatin) [81]. When control over mechanical and chemical properties is needed, then synthetic hydrogels can be used. There are notable works for a lot of synthetic hydrogels based mainly on PLA, PCL, PEG and others.

### 3.5. 4D Printing as Biofabrication Method

4D printing can be used to exploit its capabilities compared to its analogous 3D. The main difference between 3D and 4D is the nature of the material used. For the 4D, the material should have a “smart” behavior. These smart behaviors can be categorized into five main categories.

1. Shape memory (shape is changed in response to an external stimulus).
2. Self-assembly (external stimulus obligates chains into assembly in specific shape).
3. Self-actuating (Automated actuation upon exposure to an external stimulus).
4. Self-sensing (detection of external stimulus and quantification).
5. Self-healing (the damaged structure is auto-repaired)

It can be said that 4D has several advantages compared to 3D. The introduction of the fourth dimension of time, spatial and temporal control over the fabrication process and the final product makes the structure’s dynamic [42]. The 4D printing process is influenced by five main factors: (i) Type of stimulus, (ii) type of manufacturing process, (iii) interaction mechanism (stimulus and material), (iv) type of responsive material, and v) mathematical model of the transformation of the material [42]. The two areas where 4D bioprinting is mostly used in regenerative medicine and tissue engineering for fabricating complex tissue/organ geometries and controlling the tissue microarchitecture with 3D printing. 3D printing is considered as a more traditional method that can also be used for introducing cells to the structure, but 4D is a more dynamic method that can have the “smart” property of the material introduced into the structures (Figure 2).

The “smart” behavior of a material printed in 4D can be generally divided into five significant responsiveness: (i) Temperature, (ii) pH, (iii) humidity, (iv) photo, and (v) magnetism. 

Temperature responsiveness: This is one of the most frequently studied with many materials under investigation. Generally, it can be used in tissue engineering. The most common classes are shape memory polymers and responsive polymer solutions. In the first polymeric class, materials such as poly(caprolactone) dimethacrylate, soybean oil epoxidized acrylate, polycaprolactone triol (Ptriol), poly (ether urethane) (PEU), and poly (lactic acid) can be found. For the responsive polymer solutions that can be used in drug delivery and tissue engineering, polymers that can be used are poly(N isopropylacrylamide), poly(N vinyl caprolactam), gelatin and GelMA, collagen and ColMA, methylcellulose, agarose, pluronic and poly(ethylene glycol)-based block polymers [42].

pH responsiveness: This type of responsive behavior is based on polymers that are polyelectrolytes with weak acidic or basic groups. Accepting or releasing protons is the key to their responsiveness to changes in pH. Functional groups like phosphate, tertiary amine, pyridine, carboxyl, and sulfonic are responsible for changes in pH, resulting in structural changes. Drug delivery and sensing are where more applications can have these materials. Natural polymers with this responsiveness are chitosan, gelatin, dextran, alginic acid, and hyaluronic acid. In terms of synthetic ones, polymers such as poly(histidine) (PHIS), poly(acrylic acid) (PAA), poly(acrylamide/maleic acid), Poly(dimethylaminoethyl methacrylate) (PDMAEMA), Poly(2-hydroxyethyl methacrylate) (PHEMA), Sulfonamide/polyethyleneimine (PEI), Poly(N-isopropyl acrylamide-co-butyl methacrylate-co-acrylic acid) and poly(aspartic acid) (PASA) [42,82] exist.

Humidity responsiveness: Changes in humidity can alter the volume of the material. Systems with this responsiveness can transform the absorption or desorption of humidity into a driving force for movement. Polymers that can be used are poly(ethylene glycol) diacrylate (PEGDA), cellulose, and polyurethane copolymers. These materials can have applications such as stents, drug delivery, sensing, soft robotic, and tissue engineering [42,82].

Photo responsiveness: Exposure to light can undergo a physical or chemical transformation for some polymers. Generally, changes in hydrophobicity, charge, polarity, bond strength, or conformation can lead to changes in solubility, degradability, wettability, and optical properties. Using this type of responsiveness has an advantage in terms of zero contact. Polymers with specific side groups, such as diarylethenes, spiropyrans, azobenzenes, and fulgides, are commonly used. In tissue engineering, examples exist with printing hydrogels that swell or shrink upon the external stimulus of light. Some of these systems are based on PNiPAM (functionalized with spirobenzopyran) and a hydrogel of 4,4′-azodibenzoic acid, cyclodextrin, and dodecyl (C12)-modified poly(acrylic acid) [42,82].

Magnetic responsiveness: In this type of responsiveness, polymers are generally functionalized (physically entrapped or covalently bonded) with magnetic nanoparticles that have in their structure magnetic elements such as nickel, iron, cobalt, or oxides. Polymeric scaffolds prepared from these materials can undergo physical changes, structural or mechanical, as a general area of applications is more targeted to drug delivery [42,82].

Electrical responsiveness: This type of responsiveness is based on electric or mechanoelectrical external stimulus. These materials depend on their electrically conductive character to respond to the stimulus. Swelling or shrinkage of the material can undergo under an electric field. Some of these polymers can have applications in drug delivery, sensing, implant devices, artificial muscles, neural tissue engineering, and others. Hydrogels based on these types of polymers are generally based on polyelectrolytes. Some examples are sulfonated-polystyrene (PSS), poly(2-(acrylamide)-2-methylpropanesulfonic acid) (PAMPS), polythiophene (PT), poly(2-hydroxyethyl methacrylate) (PHEMA), poly(3,4-ethylenedioxythiophene): polystyrene sulfonate (PEDOT:PSS) and Poly(L-lactic acid)/Poly(3,4-ethylenedioxythiophene) (PLLA/PEDOT) [82].

## 4. Advances and Applications of Block Copolymers in 3D/4D Printing in the Area of Biomaterials

Block copolymers are a class of materials that can be used in different aspects of 3D printing. Copolymers are a class of materials with versatile properties and there are many different ways of preparation. Using copolymers instead of homopolymers in 3D printing is based mainly on the versatility and tunability of the properties the researcher wants to achieve. The temperature of the processing during 3D printing is very important. This temperature can be tuned by using a copolymer instead of a homopolymer. Crystallinity is another parameter that needs to be controlled, and copolymers can adjust this. Parameters such as wetting behavior, hydrophilicity, and solubility can also be altered by adding a more hydrophobic block or, reversely, a more hydrophilic one. Finally, mechanical properties can also be different when two or more blocks are combined. The use of copolymers uses the best properties of two synthetic polymers or polypeptides or natural polymers to adjust the material prima to easy processing during 3D printing, improved properties, and better stability and stability biocompatibility. There are numerous examples of copolymers of different architectures, such as AB, ABA, ABC, and star copolymers, or in combination with other inorganic materials or natural polymers. This review will focus on the advances and applications of biomaterials in 3D printing over the last ten years. Copolymers will be divided into (i) AB, (ii) ABA, and (iii) other architectures/combinations with inorganic materials or natural polymers.

### 4.1. AB Block Copolymers

The research in the area of copolymers type AB in the last years is mainly focused on two essential polymers that also have the lion share in the 3D printing technology, with PEG being the third one. These two polymers are poly(lactic acid) and poly(caprolactone), two polyesters with degradation properties.

Block copolymers of poly(L-lactide-co-ε-caprolactone) (PLACL), Poly(D, L-lactide-co-glycolide) (PLGA), and poly(D, L-lactide-co-glycolide) (PDLGA) were prepared via extrusion-based 3D printing in terms of extensively studying and deeply understanding how printing parameters affect degradation, printability, and properties of lactide-based medical grade polymers. In this work, authors vary printing parameters (pressure, speed, and temperature) to evaluate the relationship between composition, degradation, printability, polymer microstructure, and rheological behavior. It is crucial to notice that the innovation of this work is based on the evaluation of the polymer changes occurring during printing that many do not consider during the preparation of scaffolds. This work found that polymers had good printability at a relatively good speed and high resolution until a certain degree of degradation. Polymers of this chemical composition can thermally decompose from the first minute leading to a decrease in the average block length. PLACL had better printability at higher molecular weights with less degradation than PLGA and PDLGA, which can be explained in terms of viscosity [83].

**Figure 3:**
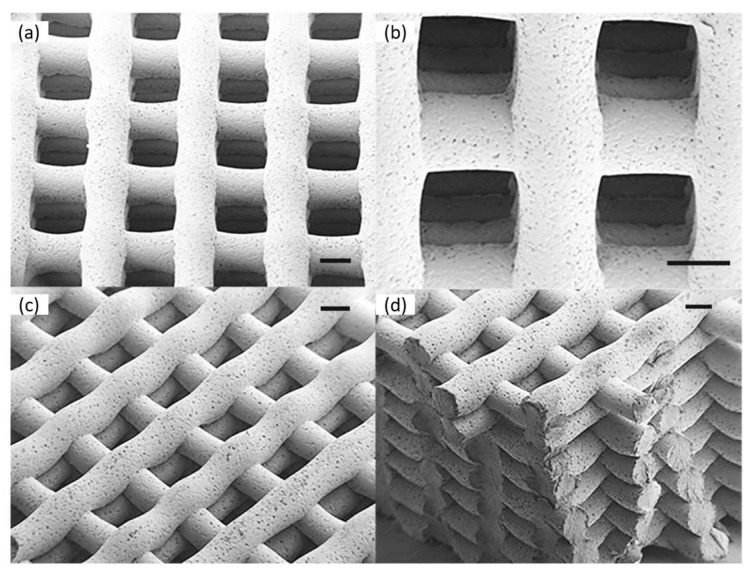
SEM images of 3Dprinted copolymer impregnated with silver nitrate scaffolds at different magnifications (**a**–**d**) (scale bars: 200 μm). Reprinted with permission from [84].

Scaffolds of PCL with poly(1,3-propylene succinate) (PCL-PPSu), including antimicrobial silver particles printed by a custom-built computer numerical control 3D printer, were prepared. These scaffolds were prepared as promising biomaterials for regenerative skin therapies. Essential points in this study are also the low-processing temperature, the enhanced degradation, and the antimicrobial properties. These scaffolds were quite porous with well-defined interconnected porosity, as seen from SEM images (Figure 3). Structures showed higher enzymatic and hydrolytic degradation rates and better hydrophilicity than PCL. Experiments with *E. coli* and *C. albicans* showed reduced microbial adhesion compared to *P. aeruginosa* and *S. aureus*. These materials can be attractive biomaterials for tissue engineering and wound healing applications [84].

**Figure 4:**
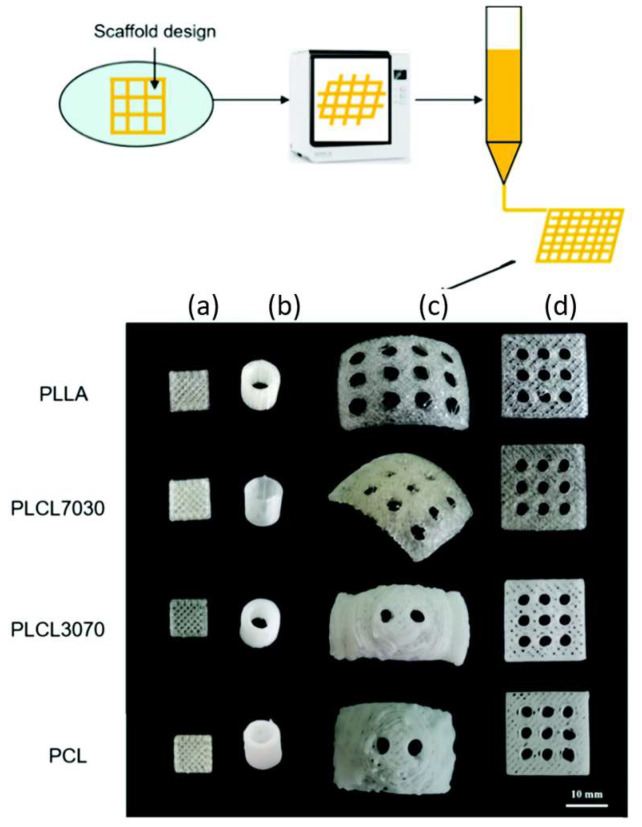
3D printed micro-structures designed by the computer-aided design model (top)and optical images of different polyester scaffolds. Reprinted with permission from [85].

Another work used poly(lactide-co-ε-caprolactone) (PLACL) as the ink for printing scaffolds with a desktop 3D printer. These scaffolds have great potential in regenerating soft tissues such as muscle, tendon, nerve, cartilage, and myocardium. An important issue is the biocompatibility and cost-effectiveness of these materials in tissue engineering scaffolds. Here, the authors promoted the incorporation of PCL into PLA as an effective strategy to control stiffness and elasticity. PCL is introduced as a solution to overcome present defects associated with PLA. Combining these two polymers can lead to adjustable mechanical properties and good biocompatibility, compared to their analogous homopolymers. Scaffolds that have different properties and different optical aspect can be seen in Figure 4 where porous cubes with or without large holes (Figure 4a,d), round tubes (Figure 4b), and cambered plates with holes (mimicking the PEEK-based cranium restoration substitute, Figure 4c) are presented [85].

PCL has also been combined in a block copolymer with polypyrrole (PPy), fabricating a 3D porous nerve guide conduit. This block copolymer was used to fabricate 3D scaffolds with an in-house built electrohydrodynamic-jet 3D printing system. These biodegradable and conductive scaffolds can lead to pretty soft structures with conductive properties and similar mechanics to the native human peripheral nerve (∼6.5 MPa). This work found improved degradation profiles to aid the growth and differentiation of the neuronal cells in vitro. These scaffolds proved to be promising materials for future use and treatment of neurodegenerative disorders [86].

**Figure 5:**
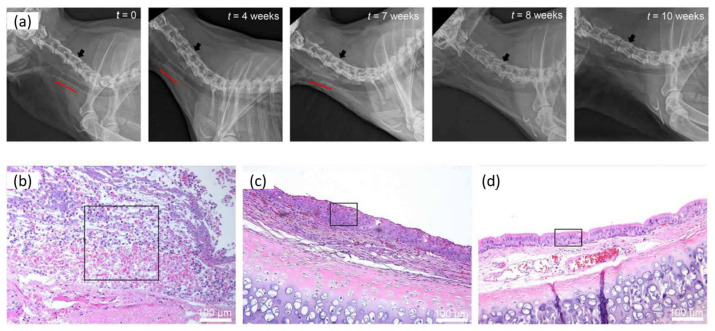
(**a**) Radiographs of a rabbit with the observation period of 10 weeks. Position of the stent is marked with a red line. Black arrows indicate the C4 vertebrae. (**b**–**d**) Inflammatory and tissue morphology changes in the rabbit’s trachea 2 (**b**), 6 (**c**), and 10 weeks (**d**) after the stent insertion. Black rectangles represent the parts of the main morphological changes over time including the area of inflammation and necrosis (**b**), squamous metaplastic epithelium (**c**), and pseudostratified columnar (respiratory) epithelium (**d**). Hematoxylin and eosin staining, magnification of 10 × 10. Reprinted with permission from [87].

Polylactide, in combination with polycaprolactone, has led to many interesting applications in the area of biomaterials. This combination of poly(D,L-Lactic Acid) (PDLLA) and PCL as photoinks led to digital light 3D printing with the DLP technique of customized and bioresorbable airway stents. This material proved to be a bioresorbable elastomer that can be used in customized medical devices where high precision, elasticity, and degradability are needed. The as-prepared stents have comparable mechanical properties to state-of-the-art silicone stents. An essential point in this work is that these stents would disappear over time, preventing the need for additional procedures. This is something that is significant for children and elderly patients. An in vivo study in healthy rabbits confirmed biocompatibility and showed that the stents stayed in place for at least seven weeks after their incorporation. After this period, they became invisible through radiography, as can be seen in Figure 5 [87].

Other materials that can be used in 3D printing with the AB architecture are also based on a thermosensitive/moderately hydrophobic poly(2 N-propyl-2-oxazine) (pPrOzi) and thermosensitive/moderately hydrophilic poly(2-ethyl-2-oxazoline) (pEtOx). By using an extrusion-based 3D bioprinter, the preparation of a bio-printable and thermoreversible hydrogel was fabricated for use in tissue engineering applications. These hydrogels have a microporous structure with high mechanical strength (3 kPa). This block copolymer was used in printing various 2D and 3D patterns with high resolution. Furthermore, hADSC stem cells were efficiently encapsulated, and the hydrogel showed cytocompatibility in post-printing cell experiments. This hydrogel can be an alternative in combination with other bioinks to improve printability or be used as a drug delivery platform [88].

Another approach was investigated with a polyester thermoplastic elastomer containing alternating semi-crystalline polybutylene terephthalate as hard segments and amorphous poly(ether terephthalate) as soft segments. This work is more orientated toward improving patient compliance and personalized drug delivery with long-acting drug delivery devices (e.g., implants and inserts). This study highlights processability and drug delivery. These 3D printed materials have outstanding mechanical properties and drug permeability similar to conventionally marketed inserts, such as intravaginal rings, using progesterone (P4) as a model drug. A significant point was the dependence of drug loading (via solvent impregnation) on the external and internal geometry of the 3D-printed structure. The drug was mainly distributed in the outer layers of large structures, hence a high burst effect during release [89].

Finally, work with blocks AB and triblock ABA based on PEG and maleic anhydride led to biocompatible hydrogels. These materials were based on PEG-diol and monomethyl ether PEG that were used to polymerize (with ring-opening polymerization) maleic anhydride and propylene oxide to AB and ABA PEG-poly(propylene maleate) (PPM) and finally to PEG-poly(propylene fumarate) (PPF). In this work, the structures were prepared via continuous digital light processing and were then photochemically printed from an aqueous solution. These 3D printed structures could potentially be applied in soft tissues, such as peripheral nerve regeneration. Experiments with three different cell types (MC3T3, NIH3T3, and Schwann cell lines) showed non-toxic behavior and compatibility [90].

### 4.2. ABA Block Copolymers

The area of block copolymers type ABA has been studied more in the last years than copolymers type AB. The polymer that is used in the majority of the cases is based on some type of PEG. The FDA considers this polymer safe and inert and has many uses in medicine, biology, and chemistry.

A material that can be used in regenerative medicine, including bioprinting, is based on the synthetic copolymer poly(propylene fumarate)-b-poly(ethylene glycol)-b-poly(propylene fumarate) (PPF-b-PEG-b-PPF). 3D printed materials via digital light processing additive manufacturing can be prepared using this copolymer. These 3D-printed amphiphilic hydrogels show the importance of the nanoscale size and ordering of hydrophobic crosslinked domains in terms of degradation and mechanical properties. It points out a direct correlation between mechanical properties and structure. Compared with other works that studied the number of cross-linking sites concerning mechanical properties/resorbability, this work also considers how these domains are connected. It was shown that size, connectivity, and phase ordering changes resulted from aggregation mechanisms driven by the length of the blocks. The importance of these nanoscale ordering can also be seen in the authors’ swelling and in vitro tests [91].

**Figure 6:**
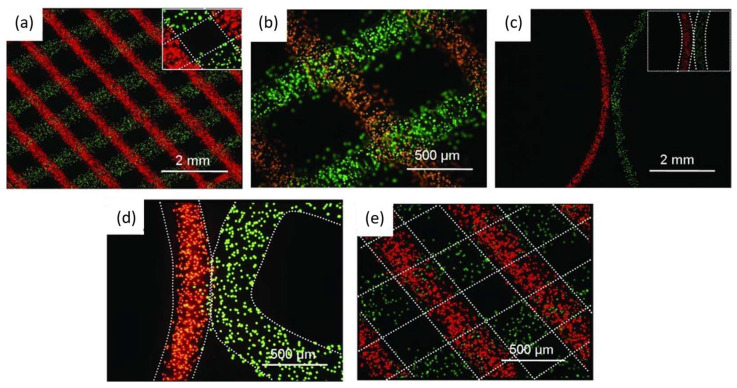
Microscopy pictures of subsequently printed layers of 25 wt% M30P10 hydrogels loaded with Dye-Trak “F” fluorescent microspheres, fluorescent lemon and fluorescent orange, at the concentration of 1 × 10^6^ spheres per mL. (**a**) Two and (**b**) three layer angled constructs (1.5 mm strand spacing) with distinct localization of fluorescent microspheres. (**c**,**d**) Printing of adjacent fibers with circular patterns with maintenance of distinct dye localization. (**e**) Two layers construct with 0.8 mm strand spacing. Reprinted with permission from [92].

In another work based on polymer poly(N-(2-hydroxypropyl)methacrylamide lactate) A-blocks, (partly derivatized with methacrylate groups), and hydrophilic poly(ethylene glycol) B-blocks (ABA) a hydrogel prepared as a synthetic extracellular matrix for tissue engineering via 3D fiber deposition [92]. According to the authors, this hydrogel can be seen as a potential applicant in bioprinting. The authors showed the preparation of 3D-printed structures with good mechanical properties, stability in photopolymerization, and well-defined vertical porosity. Mechanically, this polymer showed similarities to many natural polymers, including collagen. This similarity makes it an ideal candidate for synthetic extracellular matrix for engineering cartilage. This work has demonstrated the support of encapsulated cells until new tissues are formed, parallel with high chondrocyte viability after one and three days. The highly defined patterning of these printed materials can be observed in Figure 6, where pore shape is maintained, showing good resolution and distinct localization, after loading the fibers (layered and adjacent) with fluorescent microspheres (fluorescent lemon and fluorescent orange) [92].

An interesting work based on using an ABA copolymer as part of polyurethane is also found in the literature [93]. This work uses the poly(ε-caprolactone)-b-poly(ethylene glycol)-b-poly(ε-caprolactone) triblock copolymer (PCL-b-PEG-b-PCL) to react with hexamethylene diisocyanate to form polyurethane. These hydrogels can be used in 3D printing, especially for tissue engineering scaffolds with high fracture toughness. These structures can have excellent processability and excellent properties in terms of mechanics. The preparation of these materials was done by extrusion using a filament extruder. This work found that a high amount of water (more than 500%) can be uptaken from the materials. In terms of mechanical properties, the high elongation at break, toughness, tensile strength, and tear resistance was proven [93]. 

PEG is a polymer that is used quite often in ABA architectures. The use of PCL-b-PEG-b-PCL and PLA-b-PEG-b-PLA is another example of this. This work showed that PCL-b-PEG-b-PCL hydrogel with crystallinity could be extruded and printed with adjustments in temperature. 3D structures can effectively be prepared, and good cell compatibility was found with good mechanical properties. According to the authors, these materials can be used as a responsive hydrogel model for 3D bioprinting [94].

3D structures that can have potential applications in tissue engineering are prepared from hydrogels based on an ABA poly(isopropyl glycidyl ether)-b-poly(ethylene oxide)-b-poly(isopropyl glycidyl ether). These structures were prepared via a direct-write printer from a three-stepper motor stage. These structures can have a dual stimuli responsiveness in temperature and shear response [95].

ABA copolymers consisted of a triple stimuli-responsive poly(allyl glycidyl ether)-stat-poly(alkyl glycidyl ether)-b-poly(ethylene glycol)-b-poly(allyl glycidyl ether)-stat-poly(alkyl glycidyl ether) can create new opportunities in the biotechnological field. These materials can have three different responsiveness, based on temperature, pressure (shear-thinning), and UV light, and a direct-write printer prints them. The authors designed these triple responsiveness in order to have easy handling and transfer of the gel (temperature response), printed at ambient conditions (pressure response), and crosslinking for preparing robust structures (UV response). By changing the composition of these materials, the properties of the final object can be tuned, leading to an expansion of a set of materials [96].

An ABA copolymer was prepared from PEG (Block B) and partially methacrylated poly[N-(2-hydroxypropyl) methacrylamide mono/dilactate] (Block A). These hydrogels could be attractive biomaterials since they have biodegradability, tunable thermoresponsiveness/mechanical properties, and cytocompatibility. To improve printability, mechanical properties, and long-term stability, the authors have incorporated methacrylated chondroitin sulfate (CSMA) or methacrylated hyaluronic acid (HAMA). This work showed that the pure ABA copolymer laden with equine chondrocytes showed potential for significant cartilage-like tissue formation in vitro. Finally, incorporating HAMA or CSMA resulted in 3D porous structures with excellent cell viability and improved properties. Overall, are attractive systems for the design of 3D cell-laden constructs for cartilage regeneration [79].

The last work based on PEG as the main component is based on a triblock with PCL as the A block. These hydrogels showed tunable mechanical properties, mainly for soft tissues and high elasticity. Good compatibility with cells to support fibroblast growth in vitro was also studied. The most crucial point of this work is the bioprinting of cells with these hydrogels to form constructs of cell–gel with high viability. The printing procedure has the main component, and a dispenser is mounted onto a robotic stage; then, a motor-controlled stage acts as the printing substrate. A syringe pump drives the extrusion-based dispenser. This work has demonstrated a system with tunable properties, elasticity, and biodegradability. According to the authors, this hydrogel may be fully compatible with other biomaterials, such as proteins, growth factors, peptides, or other bioactive molecules [97].

Apart from PEG as the main component of ABA copolymers, another work involves the acrylic ABA, composed of poly(methyl methacrylate) (PMMA) as block A and poly(n-butyl acrylate) (PnBA) as block B. This copolymer is categorized as a thermoplastic elastomer and is for industrial use. This material is to be used as material in the dental field. The results showed that this copolymer has good physical properties, concluding that it can be used to make provisional restorations for dental 3D printers [98].

**Figure 7:**
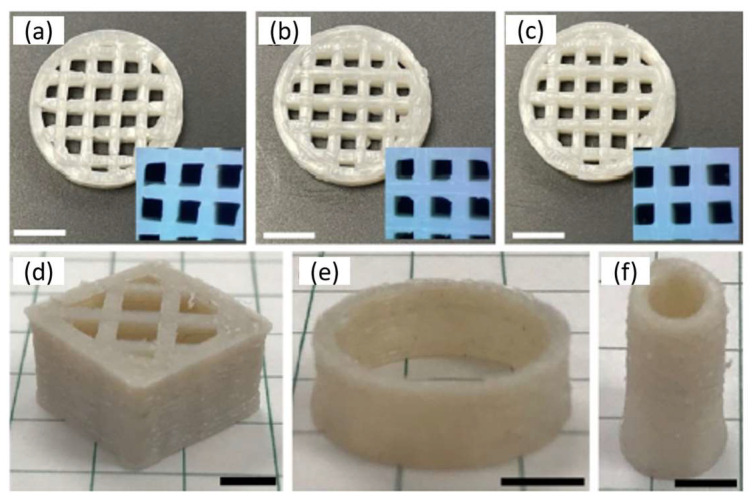
(**a**–**c**) Photos of 3D-printed cylinders with infill patterns from the SIBS/PS blends: (**a**) SIBS/PS (77/23), (**b**) SIBS/PS (67/33), and (**c**) SIBS/PS (57/43). The insets show higher-magnification images taken with an optical microscope. (**d**–**f**) 3D-printed objects with different shapes from SIBS/PS (57/43). Scale bars are 5 mm. Reprinted with permission from [99].

An interesting work for materials potentially used as medical implants where biocompatibility and stability are essential is based on a copolymer of poly(styrene-b-isobutylene-b-styrene) (SIBS)/PS homopolymer blend. In this work, filaments of the blends were used for FFF (Fused filament fabrication) 3D printing. 3D constructs can be prepared, as can be seen in Figure 7. The use of PS homopolymer is based on increasing the toughness of the SIBS copolymer due to its limitation in printing because it is soft. This work found that the microarchitecture is tunable, and printability is excellent under specific conditions [99].

**Figure 8:**
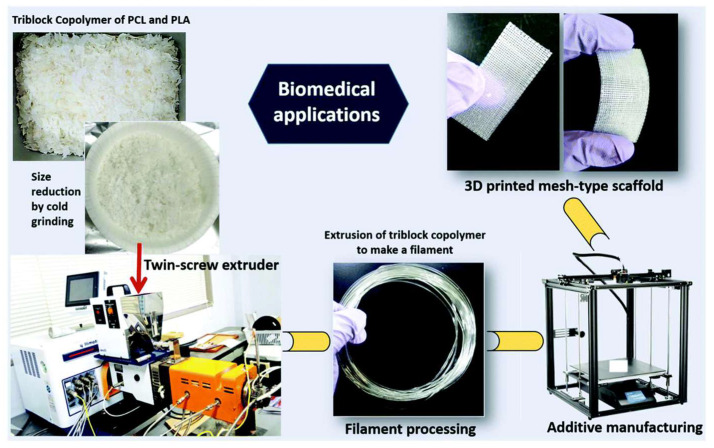
Melt processing of the triblock copolymer (synthesized by the scale-up method) to fabricate the filament which was successfully used for 3D printing a mesh-type scaffold. Reprinted with permission from [100].

A triblock that can be prepared in large quantities is investigated in another work with polymers first processed from a twin-screw extruder for making the filament and then 3D printed (Figure 8). This polymer is based on PCL as the middle block (B) and PLLA or PDLA as the A blocks. The prepared 3D constructs were tested for biocompatibility via MTT assay, using rat bone osteosarcoma cells (UMR-106) to evaluate them as potential biomaterials. It was found that the block length and the composition of the ABA affect the macroscopic properties of the specimens and the mechanical ones. This is important since, varying the final material’s properties, customized constructed can be prepared. Finally, the MTT assay found a non-toxic nature of the material [100].

An interesting work was proposed using an ABA triblock based on PCL-b-PTMC-b-PCL (trimethylene carbonate as TMC) [101]. The idea of this study was to prepare copolymers with biodegradability and to evaluate physical properties and potential melt-processable thermoplastic elastomeric biomaterials in 3D printing via extrusion of the polymer solution. Mechanical properties showed a tensile strength of 120 MPa, elongation at break 620%, and tensile strength of 16 MPa. With melting points close to 58 °C, these materials can be extruded and are promising as biomaterials for use as implants and scaffolds for tissue engineering. The advantage of these materials is the microporosity that can be tuned for cell culture; however, cell compatibility must be evaluated [101].

**Figure 9:**
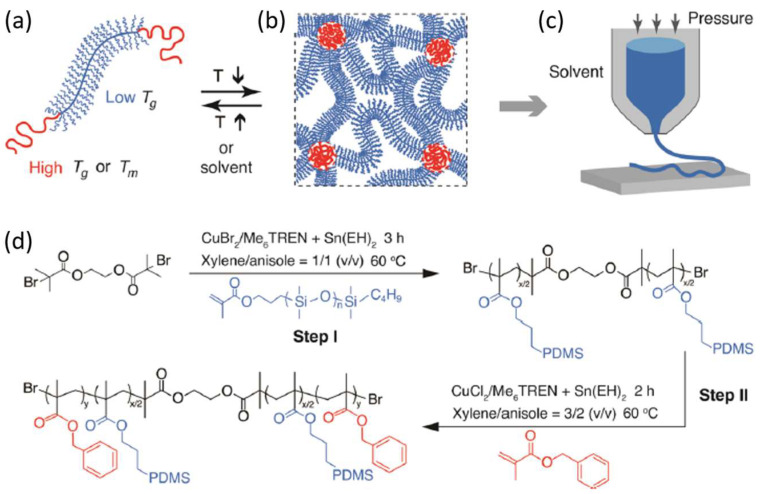
Design concept and synthesis of 3D printable, reversible, ultrasoft, and stretchable elastomers. (**a**) Schematic of a responsive linearbottlebrush-linear triblock copolymer. (**b**) At low temperature, the middle bottlebrush blocks (blue) act as elastic network strands, whereas the high Tg end linear blocks aggregate to form spherical glassy domains. (**c**) Glassy domains dissociate at high temperature or in the presence of solvents, resulting in a solid-to-liquid transition of the network. The stimuli-triggered reversibility allows the elastomers for direct-write 3D printing. (**d**) Synthesis of linear-bottlebrush-linear triblock copolymers using ARGET ATRP. The side chain of the middle bottlebrush block is linear polydimethylsiloxane (PDMS), whereas the end blocks are linear poly(benzyl methacrylate) (PBnMA). A bottlebrush-based triblock polymer is denoted as BnMAy-b- PDMSxw-b-BnMAy, in which y is the number of repeating BnMA units, x is the number of PDMS side chains per bottlebrush, and w represents theMW of PDMS side chains in kg/mol. The weight fraction of the end blocks in the triblock copolymer is kept below 6% to ensure that the bottlebrush-based ABA triblock copolymers self-assemble to a sphere phase. Reprinted with permission from [102].

**Figure 10:**
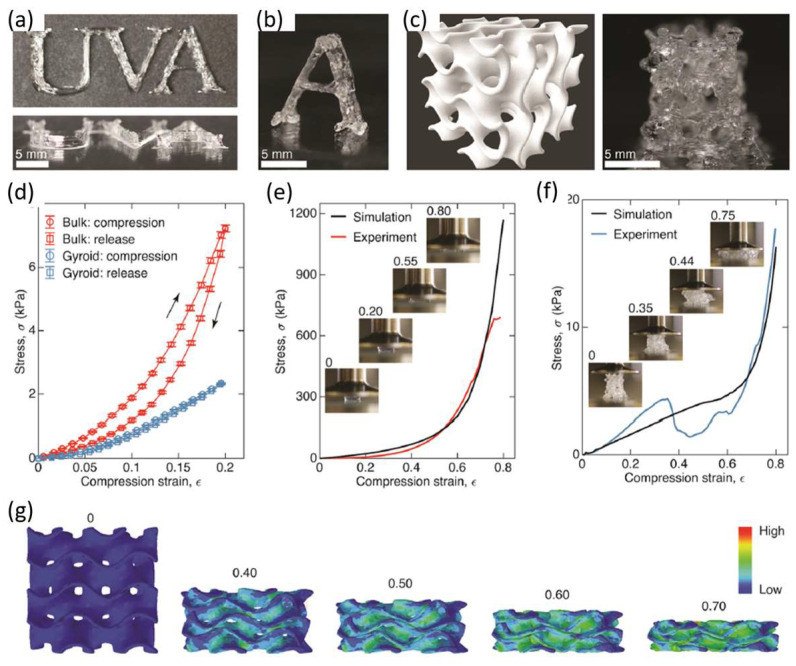
Direct-write printing soft elastomers to create deformable 3D structures. (**a**) 3D printed UVA initials with a stack thickness of 2 mm. Upper: bird’s eye view; lower: side view. (**b**) Free-standing, 3D printed letter “A”. (**c**) 3D rendering of a cubic gyroid (left) and the corresponding printed product with dimensions 10 × 10 × 10 mm^3^ (right). (**d**) For the bulk sample, the compression−release profile exhibits a hysteresis associated with 23% energy dissipation (dashed lines), whereas for the gyroid, there is almost no energy dissipation, as evidenced by the complete overlap between the compression and release profiles (solid lines). The apparent Young’s modulus, Ε = σ/ε, of the gyroid is about 8 kPa, nearly half of 20 kPa for the bulk; this is likely because the porous gyroid has a lower density of about 1/2 of the bulk. The strain rate is 0.005/s. Error bar: standard deviation for *n* = 5. (**e**) As the compression strain increases from 0.2 to 0.55, the bulk sample exhibits strain-stiffening with the compression stress increasing from 8 to 130 kPa, and further compression with ε > 0.55 results in material fracture (optical images). The stress−strain profile is used to calibrate the FEA simulation (dashed line). (**f**) Cubic gyroid exhibits a nearly linear elastic deformation up to ε = 0.35, at which the stress is about 5 kPa, 10 times lower than the 50 kPa for the bulk. Slightly above ε = 0.35, the stress exhibits a sharp decrease (solid line). (**g**) Decrease is associated with structural collapse of the gyroid, as indicated by comparing the snapshots from FEA simulation (dashed line in (**f**) and upper panel) with the optical images of the gyroid (lower panel) under various extents of compression. The use of UVA initials in 3D printing is under the permission from the UVAOffice of Trademark and Licensing. Reprinted with permission from [102].

A triblock ABA copolymer based on polydimethylsiloxane (PDMS) (B block) and poly(benzyl methacrylate) (PBnMA) (A block) was prepared as a linear-bottlebrush architecture (Figure 9). This copolymer could have much potential in future applications since it has extraordinary properties such as stimuli-reversible, extraordinarily soft, and stretchable elastomer (Figure 10). This material can be used as ink for direct writing in 3D printing structures without post-treatment or external mechanical support. This work compares the material used with existing 3D printable elastomers, showing more than two orders of magnitude softer material and six times more strechable. The authors explain these results from the bottlebrush molecular architecture, which prevents entanglement formation, whereas stretchability ought to have an extensive network strand size. These elastomers can be readily used as a matrix for functional nanoparticle-polymer composites. The self-assembly and thermoreversibility can be an essential advantage for direct-writing printing to avoid solvent evaporation-induced material defects during printing [102].

Finally, a material that can have potential use as bioink is studied in another work, with the use of ABA triblock hydrophilic poly(2-methyl-2-oxazoline) (pMeOx) (block A) and a more hydrophobic poly(2-iso-butyl-2-oxazoline) (piBuOx) (block B). Hydrogel scaffolds of a 20% aqueous solution were printed with a compact bench-top 3D bioprinter from these materials. Rheological experiments show a soft hydrogel above 25 °C with an elastic modulus of 150 Pa, while an increase at almost 600 Pa is observed at 37 °C. The significant point of this work towards the use as bioink was the low cytotoxicity after 24 h at 37 °C observed for both HEK and Calu-3 cells. This work indicates that the fabricated hydrogel may be a potential candidate for printable, functional bioink [103].

### 4.3. Other Architectures of Block Copolymers and Systems with Other Materials

The preponderance of the works in block copolymers consisted of ABA and AB architectures. Most polymers are based on PLA, PCL, and PEG. In the literature, the last year’s other architectures based on the same polymers as AB/ABA architectures or other polymers are currently under investigation. Moreover, using natural polymers, polypeptides, hydroxyapatite, or clay has contributed much to 3D printing in the last few years [104,105,106,107,108,109,110,111,112,113,114,115,116,117,118,119].

#### 4.3.1. Other Architectures of Block Copolymers

**Figure 11:**
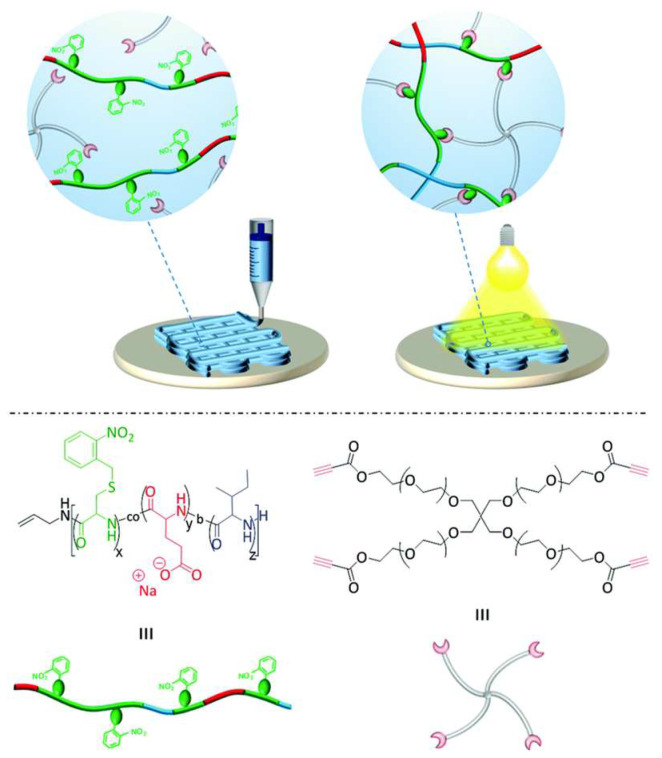
Schematic showing photocleavage of nitrobenzyl protecting groups revealing free thiol functionalities that react with peripheral propiolate functional groups on four-arm PEG. Reprinted with permission from [104].

The use of polypeptides as alternative polymers in 3D printing is also explored [104,108]. This work involves the preparation of highly stable hydrogels with crosslinking under UV. The triblock is an ABC consisting of peptides, glutamic acid, nitrobenzyl-protected cysteine, and isoleucine. This polymer then is deprotected, and a reaction of the cysteine residue with alkyne functionalized four-arm polyethylene glycol (PEG) via nucleophilic thiol–yne chemistry occurred (Figure 11). These hydrogels showed remarkable shear-thinning properties according to the authors. Very important is gellation at lower concentration (3 wt%), desired mechanical properties, low cytotoxicity, and high printability. This material can be an alternative catalyst-free curing method for 3D-printed structures for different biomedical applications [104].

**Figure 12:**
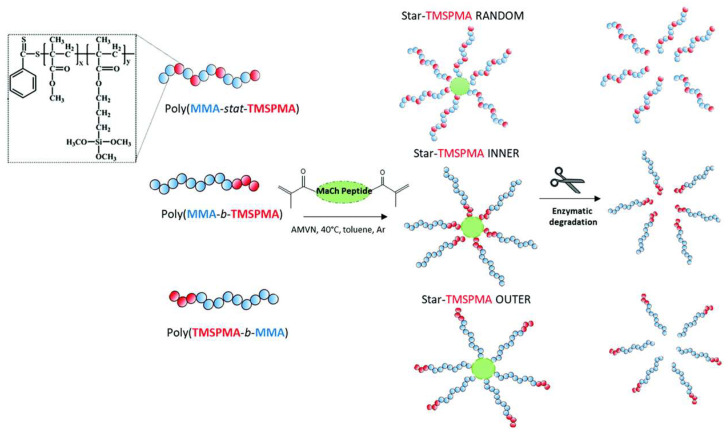
Schematic illustration of the poly(MMA-TMSPMA) star polymers synthesised with arms of three different architectures (random, inner and outer) crosslinked by an MaCh-peptide core and cleavage by collagenase activity. Reprinted with permission from [105].

A similar architecture was also investigated to create enzyme-cleavable inorganic-organic hybrid inks with potential applications in scaffolds for bone regeneration. The copolymer used was based on 3-(trimethoxysilyl)propyl methacrylate (TMSPMA) and methyl methacrylate (MMA). The arms were connected at a core consisting of degradable enzymes using a collagenase peptide sequence GLY-PRO-LEU-GLY-PRO-LYS. Three-star copolymers were prepared TMSPMA was randomly distributed, or inner block or outer block (Figure 12). The inorganic-organic hybrid was prepared of a composition of 70 wt% polymer and 30 wt% silica via the sol-gel method and was used for direct 3D extrusion printing without additional carriers or binders. From this work, it was shown that the 3D constructs could be degraded from endogenous tissue-specific enzymes that are involved in the natural remodeling of bone. The specific position of the TMPSMA group controlled the hybrid formation, mechanical properties, degradation, and printability. Better results were found for the inner-star TMSPMA, which led to a more flexible and tougher material than the others [105].

A different approach in terms of architecture comes from a work where grafted copolymer was prepared [106]. In this work, a PEDOT-g-PCL (poly(3,4-ethylendioxythiophene)-g-poly(ε-caprolactone)) synthesized intending to fabricate electroactive scaffolds for muscle tissue engineering (bioelectronics). Here, it was found that the percentage of PEDOT is a crucial parameter in printability and that only low percentages led to the process with direct ink writing. Biocompatibility of the materials was evaluated with 8220 muscle cells showing encouraging results in compatibility, cell alignment, and myotubes differentiation [106].

Hydrogels that can be used as bioinks with dual sensitivity are very important and can have many potential applications, especially in tissue engineering [107]. In this way, via an microextrusion bioprinter, a triblock copolymer consisted of poly(lactide-co-glycolide)-b-polyethylene glycol-b-poly(lactide-co-glycolide) with acrylate groups in the chain is reported. This hydrogel proved thermo/photo sensitive with excellent shear-thinning properties and fast recovery in the elastic region. The 3D printed scaffolds were very stable after the photopolymerization, which can lead to low-cost and mass production on an industrial scale [107].

Another work of star architecture is based on an amphiphilic copolymer of poly(benzyl-L-glutamate)-b-oligo(L-valine), which forms hydrogels via hydrophobic interactions [108]. This star copolypeptide can be used as ink to rapidly fabricate defined microstructures, opening the road for manufacturing complex scaffolds, which is far more difficult with conventional methods. The fabricated structures were degradable, did not affect the metabolic health of fibroblasts (cell line Balb/3t3), and could have a favorable release of encapsulated molecules [108].

**Figure 13:**
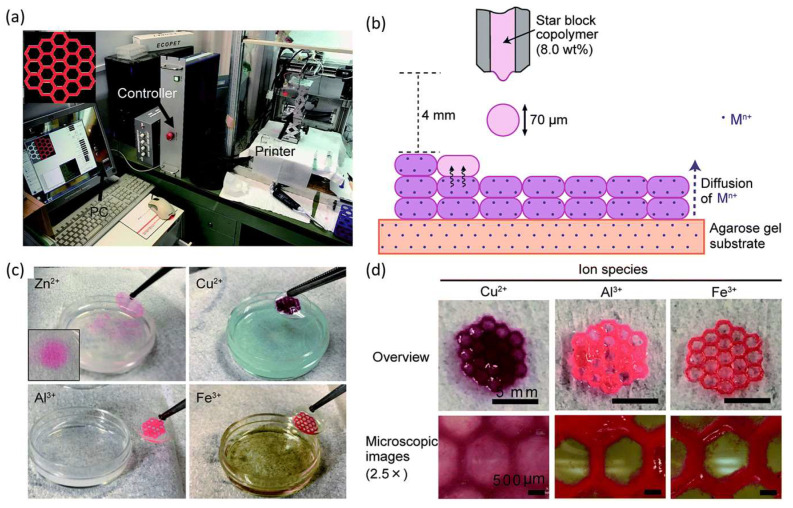
3D inkjet printing of the star block copolymer hydrogels crosslinked using metallic ions. (**a**) Overview of the inkjet printing system. Inset: horizontal slice of the design of the 3D hydrogel structures that was inputted into the printer. (**b**) Schematic illustration of the printing process. The star block copolymer solution was ejected from the inkjet nozzle towards the agarose gel substrate containing metallic ions. The printed droplets show gelation layer-by-layer through metallic ions supplied from the substrate. (**c**) Transfer of the 3D-printed hydrogels from the substrate. Inset: the gel printed on the substrate containing Zn^2+^ showed extremely low resolution. (**d**) Overview and microscopic images of the 3D-printed hydrogels crosslinked using Cu^2+^, Al^3+^, or Fe^3+^. The hydrogel crosslinked using Zn^2+^ is not shown because it was too brittle to maintain its structure when transferred from the substrate. Reprinted with permission from [109].

A different approach in the star architecture was introduced by another work, using a dendritic polyester core with a poly(oligo(ethylene glycol) methyl ether acrylate) inner layer and a poly(acrylic acid) as the outer layer [109]. The solution formed a hydrogel by adding metallic ions (zinc, copper(II), aluminum, and ferric ion). 3D structures were printed using a custom-made inkjet printer (Figure 13). It has been reported that the fast gelation of the star upon the addition of the ions can give a potential candidate that can be used in 3D inkjet printing through an in-process cross-linking approach. The authors showed that an 8% polymer solution has enough viscosity to be ejected from the inkjet nozzle. Adding Zn^2+^, Cu^2+^, Al^3+^, and Fe^3+^ formed different hydrogels that also altered the dynamic viscoelasticity. The highest resolution upon printing was found for hydrogel with Fe^3+^ [109].

**Figure 14:**
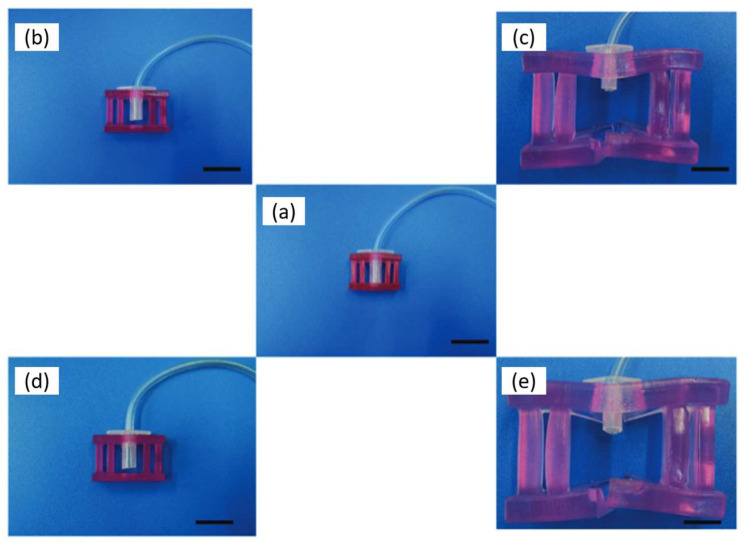
3D printed valve structure of FA70(80) at different conditions. (**a**) Dry, (**b**) pH 2.0/37 °C (**c**) pH 7.4/37 °C, (**d**) pH 2.0/6 °C and (**e**) pH 7.4/6 °C. (Bar: 2 cm.). Reprinted with permission from [110].

An interesting approach is based on the Pluronic F127, poly(ethylene oxide)-b-poly(propylene oxide)-b-poly(ethylene oxide) (PEO–PPO–PEO) with modified end groups of dimethacrylate (FdMA). This component is responsible for reverse thermo-responsiveness; the second is acrylic acid for pH responsiveness. In that way, the FdMA-co-acrylic acid hydrogels will have dual responsiveness. 3D structures via stereolithography are formed, and their environmentally sensitive dimensional behavior was evaluated. As expected, it was found tunable responsiveness in terms of temperature and pH. Combining hydrogels with different compositions, a different response of the structures obtained in temperature and pH, altering their size and geometry accordingly (Figure 14). The novel structures that were 3D printed are expected to contribute to the field of medical devices since they can change their size-volume-geometry “on command”, by changes in the temperature and pH [110].

Finally, the pluronic F127 (PEO–PPO–PEO) was used as pentablock, with the end blocks being PLA or PCL. These pentablocks were also modified with 2-isocyanatoethyl methacrylate to improve printability and can be used with acrylic acid and form hydrogels. These structures could have dual responsiveness of pH and temperature due to their blocks and degradability because of PLA and PCL. Adjusting the composition of PLA/PCL, the physicochemical properties of the hydrogels can be tuned, such as water absorption and biodegradation. These structures showed a fast and reversible swelling–deswelling response in pHs 2.0–7.0 or between 10 to 37 °C. The dual behavior for BSA release at different rates has been exploited by changing the release conditions. It has been shown that the PLA/PCL ratio plays an important role. It has been suggested that these materials can be good candidates for colon protein release formulations [111].

#### 4.3.2. Block Copolymers with Other Type of Materials

Research in block copolymers and other materials also has significant and interesting publications [112,113,114,115,116,117,118,119]. Work preparing bioinks for hydrogel implants for controlled drug release is based on different formulations. These systems fabricate cross-linkable chitosan with PLA-PEG and PEGDA. The authors are presenting three different bioink systems: (i) Methacryloyl chitosan/PEGDA, (ii) PLA-PEG (micelle)/PEGDA, and (iii) methacryloyl chitosan/PLA-PEG (micelle)/PEGDA, which are all systems photo-crosslinked during 3D printing. This work studied the biocompatibility–biodegradability of the systems for the controlled release of the hydrophobic drug simvastatin from 3D structures hydrogels for osteogenic stimulation. Tuned mechanical properties and swelling behavior was found for the different systems. The viability of these hydrogels was proved when in contact with NIH3T3 fibroblast cells. The critical point of this work was increased release over 14 weeks with a therapeutic concentration [112].

Another work is based on the preparation of polyurethane, with one of the components being an ABA type or ABCBA pentablock [113]. The first case is PCL-PEG-PCL, and the second one is PLA-PCL-PEG-PCL-PLA. In this way, the final product will also have a biodegradability property that can have vast wide-range utility in various applications as scaffolds. The different compositions and lengths of the blocks led to tunable biodegradability, elastic properties, hydrophilicity, morphology, water uptake, and thermal properties [113].

A work based on combining organic-inorganic components is also found in the literature [114]. This work focuses on a thermoplastic amphiphilic poly(lactic acid-co-ethylene glycol-co-lactic acid) combined with hydroxyapatite. This work aims to create scaffolds with shape-memory effects and macroporosity that can be fabricated by rapid prototyping. These systems were found to have a shape-memory effect at a safe triggering temperature of 50 °C and high elasticity at room temperature. The incorporation of hydroxyapatite until 20% increased the tensile modulus while maintaining shape-memory properties at more than 90%. All the above led to the conclusion that these systems showed that osteoconductivity and osteoinductivity make them ideal candidates for bone regeneration [114].

**Figure 15:**
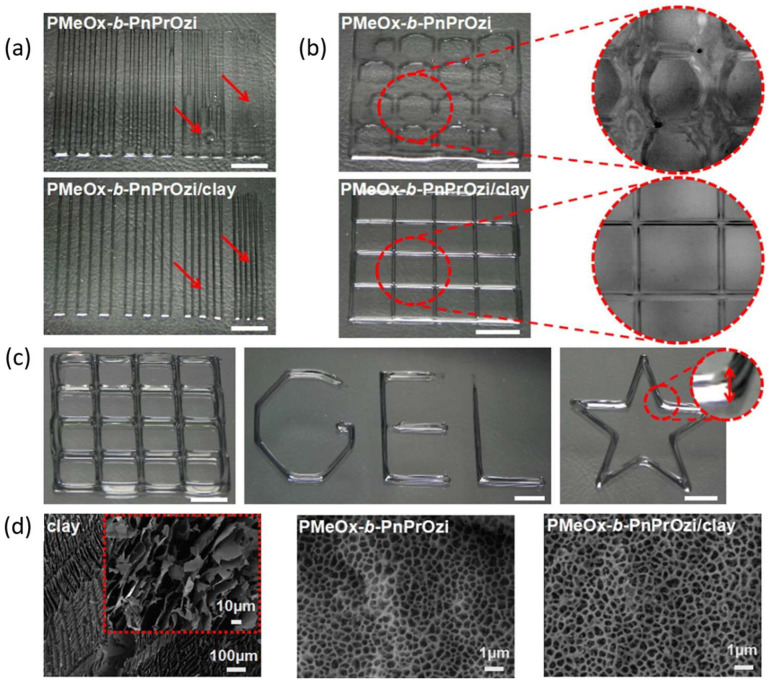
(**a**) 2D printed patterns for assessing the minimal strand-to strand distance and shape fidelity of biomaterial inks (red arrows are guiding for eyes). (**b**) Optical and stereomicroscopic images of the printed constructs composed with three layers of 5 × 5 orthogonal strands with a base area of 20 × 20 mm^2^ (amplified in dashed red circles). (**c**) Photographic images of 3D printed six layers of a 5 × 5 woodpile structure, letters ‘‘GEL’’ and a five-pointed star with PMeOx-b-PnPrOzi/clay hydrogel. (**d**) SEM images of clay and cryo-SEM images of PMeOx-b-PnPrOzi and PMeOx-b-PnPrOzi/clay. Scale bars in (**a**–**c**) represent 5 mm. Reprinted with permission from [115].

Another organic-inorganic system is based on the thermoresponsive block copolymer poly(2-methyl-2-oxazoline)-b-poly(2-n-propyl-2-oxazine) (PMeOx-b-PnPrOzi) combined with nanoclay laponite [115]. The work wants to show this formulation’s potential application as bioink and evaluate the critical properties relevant to extrusion bioprinting. The authors could show the system’s thermoresponsive character and enhanced viscoelastic properties, leading to improved printability (Figure 15). According to the authors, this material can be a versatile support bath for fluid extrusion printing, thermoresponsive self-protection, controlled drug delivery, and a sacrificial template in microfluidic devices [115].

In order to increase the mechanical properties of polymers to meet the requirements of 3D bioprinting by extrusion, this work proposes the addition of rigid nanoparticles of cellulose nanocrystals (CNC) into a poly(ε-caprolactone/lactide)-b-poly(ethylene glycol)-b-poly(ε-caprolactone/lactide) (PCLA-PEG-PCLA) triblock copolymer. The results showed increased thermal stability, a broader gel state (in terms of temperature) and increased mechanical properties with CNC at higher concentrations. This work proved that incorporating rigid CNC into amorphous gels could improve printability [116].

**Figure 16:**
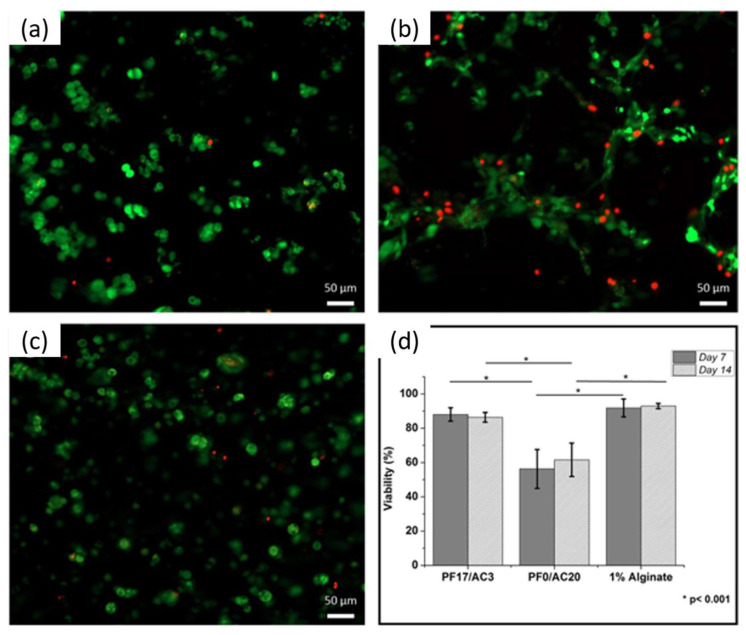
Cell viability of encapsulated bovine chondrocytes at day 14 with live cells in green and dead cells in red. (**a**) Nanostructured PF17/AC3 (**b**) PF0/AC20 and (**c**) 1% alginate. (**d**) Viabilities of encapsulated chondrocytes at day 7 and day 14. Reprinted with permission from [117].

In another work [117], the authors have a different approach based on nanostructuring in order to increase the biocopmatibility of pluronic gels at printable concentrations. This work fabricates 3D constructs based on the mixing of acrylated and non-acrylated pluronic F127 with hyaluronic acid via UV crosslinking. These gels have a reversible thermo-gelling property and good rheological properties due to F127. A very important point is also the 14 day cell viability that was assessed using a live/dead assay (viable cells stained green and dead cells stained red fluorescent), as shown in Figure 16 [117].

To prepare a hydrogel suitable for cartilage 3D printing applications, a material based on methacrylated chondroitin sulfate and a thermo-sensitive poly(N-(2-hydroxypropyl) methacrylamide-mono/dilactate)/polyethylene glycol triblock copolymer was prepared [118]. The 3D technique for preparing these materials is based on a bioprinter equipped with UV lamps. These hydrogels showed superior rheological properties when compared to their homopolymer analogous. This polymer solution was used as ink to fabricate 3D structures with different porosity. An important point was the good survival and proliferation of chondrogenic cells when incorporated into the hydrogel [118].

Finally, an exciting work involving a two-component hydrogel for preparing scaffolds is presented here [119]. The first component of hydrogel consists of an ABC copolymer in combination with hyaluronic acid (HA) or PEG, both functionalized with N-hydroxysuccinimide. The ABC copolymer was a PEG-b-NiPAAm-b-HPMACys (N-2-Hydroxy-propyl)methacrylamide-Boc-S-acetamidomethyl-L-cysteine) and was used to pre-crosslinked with oxo-ester mediated native chemical ligation and physically crosslinked after deposition on a 37 °C printing plate. The researchers proved that the significant increase in Young modulus (17 to 645 kPa) by covalently grafting to a thermoplastic polymer scaffold (Poly(hydroxymethylglycolide)-co-ε-caprolactone). The authors have successfully demonstrated the increase in mechanical properties of the two-component hydrogels and also high cell viability of chondrocytes in the structure with hyaluronic acid [119].

## 5. Challenges and Future Works

3D printing, alongside 4D, is currently in constant evolution. New materials, techniques, and 3D structures for various applications are discovered yearly. The future of this technique in the biomaterials area has great potential but also many challenges and obstacles that need to be considered, improved, or changed to the level of the different applications required.

### 5.1. Challenges

Regarding challenges, the three main directions that need to be considered are: (i) Materials and techniques, (ii) scaffold architecture, and (iii) cell viability/vascularization.

(I) Materials and Techniques

The choice of material is a very crucial issue to deal with for scientists working with 3D printing. The chemical properties, physical properties, potential stimulus, printability, versatility, cost, degradability, and bioavailability will come from the selection of the materials. For hydrogels, for example, the printing resolution needs to be considered. Now it is at 0.3 mm, and for specific scaffolds, a better resolution will be required. Degradation or not is another challenge, especially for scaffolds, where new cells must be adhered to and slowly replaced by the printed scaffold. In the case of by-products after degradation, a careful evaluation must be done to ensure that they are not toxic and do not affect the environment where the 3D-printed object was present. Mechanical properties play a vital role since they are responsible for the printability and stability of the structure. Different parts of the human body need other mechanics for the constructs. In part of the materials, the biocompatibility must always be considered, and the way of sterilization is also a crucial issue for the choice of material.

(II) Scaffold architecture

In the case of scaffold architecture, the physical characteristics should be improved, meaning pore size, distribution and morphology, and topography. It should always be taken in mind that in these scaffolds, cells will adhere and grow and substitute the existing structure. All physical properties should be adequate for the specific cells. During 3D printing, many hydrogels show inhomogeneity due to the uncontrollable crosslinking, leading to poorer mechanical properties, so the way crosslinking is to happen during the preparation of the scaffolds is also quite important.

(III) Cell viability/vascularization

Finally, there are still many unsolved issues in terms of cell viability and vascularization. Cells must be viable and evenly distributed onto the scaffold in tissue engineering. Cells loaded to the printer are also crucial since viability is important during loading, printing, and postprocessing. At present, existing 3D printing technologies can be used to precisely dispense one or two cell-laden hydrogels for the construction of simple or vascularized tissues. They need to recapitulate the intrinsic complexity of vasculature in a natural organ. Building a real blood circulatory network is challenging and one of the future directions. The last point is the 3D printing of personalized constructs or drug delivery for each patient [41,76,77,82].

### 5.2. Future Works

Overcoming the challenges that now exist in 3D printing technologies, the future can be fruitful for functional materials in biology and medicine. The main lines of investigation that the researchers will focus their research on will be:

(I) Materials
(II) Combination of natural and synthetic polymers
(III) 3D printers
(IV) Crosslinking
(V) Testing and simulation of the 3D structures
(VI) New techniques of processing incorporated in 3D

The selection of materials will always be an area of investigation that can lead to the discovery of new combinations or polymers. These formulations will affect the processability, degradation, mechanical properties, and cell viability. Controlling the diversity and the different polymers (natural or synthetic), desirable physiological functions will be captured in specific micro/macro-environments. An investigation will also be made into the 3D printers to improve their capabilities, especially in the bioprinting technology. 

In crosslinking, new techniques will be studied to differentiate pre and post-printing chemistries that can improve and better control the crosslinking. In many formulations, the material to be printed consist of different components that can create formulations with agglomerations; therefore, new methods that ensure homogeneous distribution of the different phases must be studied. The testing and simulation of these 3D constructs is also significant. In biomaterials, high standards must be applied in terms of mechanical, physical, chemical, and stimuli. Finally, the investigation will be done with other techniques, such as electrospinning, biofabrication, microfluidics, and 4D printing [41,76,77,82].

## 6. Conclusions

In the last 10–12 years, 3D/4D printing has changed dramatically. Techniques or polymers used at the beginning are modified or functionalized to have better printability, processability, resolution, and dual properties with or without stimuli. Researchers are constantly investigating new materials and ways of preparing 3D constructs. Every time they fabricate materials that can substitute hard or soft tissues, used as drug delivery vehicles, scaffolds where cells will grow and proliferate and structures printed alongside different types of cells. The structures are every time more complex and try to mimic organs or systems or functions of the human body.

**Figure 17:**
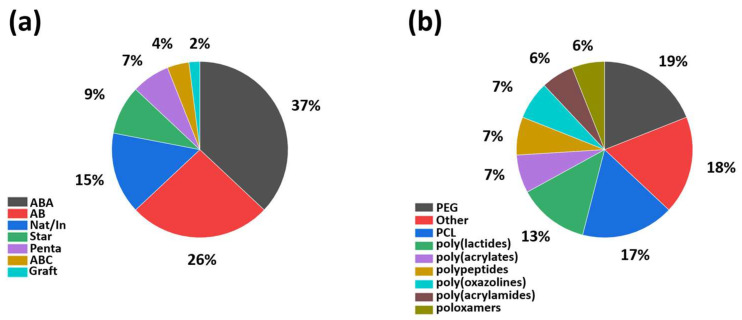
(**a**) Pie chart of the different architectures of block copolymers investigated the last years, and (**b**) pie chart of the different polymers used in block copolymers in the last years.

Someone will ask, where is the limit? It can be said that it is almost infinite. Next-generation printed devices are expected to respond after sensing first local changes in live tissues (infection, cancer, neurodegeneration, or inflammation), offering more personalized therapy. In the case of polymers, many architectures and polymers are already in use. Apart from homopolymers (synthetic or natural), block copolymers play a significant role in the evolution and development of 3D/4D printing. Figure 17 shows two graphs of the different architectures and polymers of block copolymers used in the last 10–12 years. Most of the architectures are based on AB and ABA but with natural polymers (e.g., chitosan or hyaluronic acid) or inorganic materials (e.g., hydroxyapatite or clay) and star-like polypeptides to gain their way in more and more applications. In terms of polymers, poly(ethylene glycol), poly(caprolactone), and poly(lactide) are used in half of the works in one way or another. Nevertheless, new polymers are used in at least 18% of the works. One-third of the works also use candidates with potential due to their properties, such as acrylates, acrylamides, peptides, poloxamers, and oxazolines.

Finally, it has to be said that the future will bring many discoveries in smart materials for 4D (bio)printing, complete automations in high-resolution printers, computational modeling, and artificial intelligence. The therapy will be transformed into a more personalized one thanks to 3D printing and its constant evolution.

## Data Availability

Not applicable.

## Abbreviations

2PP	two-photon polymerization
AB	block copolymers
ABA	triblock copolymers
ABC	triblocl terpolymers
AJP	aerosol jet printing
CLIP	continuous liquid interface production
CNC	cellulose nanocrystals
CSMA	methacrylated chondroitin sulfate
DIW	direct ink writing
DLP	digital projection lithography
EHD	electro-hydrodynamic printing
FDM	fused deposition modeling
FdMA	pluronic F127 dimethacrylate
FFF	fused filament fabrication
HAMA	methacrylated hyaluronic acid
HAMA	hyaluronic acid
HPMACys	N-2-Hydroxy-propyl)methacrylamide-Boc-S-acetamidomethyl-L-cysteine
PAA	poly(acrylic acid)
PAMPS	poly(2-(acrylamide)-2-methylpropanesulfonic acid)
PASA	poly(aspartic acid)
PBF	powder bed fusion
PBnMA	poly(benzyl methacrylate)
PBT	poly(butylene terephthalate)
PCL	polycaprolactone
PCLA	poly(ε-caprolactone/lactide)
PCL-PPSu	poly(1,3-propylene succinate)
PDLGA	poly(D, L-lactide-co-glycolide)
PDMAEMA	poly(dimethylaminoethyl methacrylate)
PDMS	polydimethylsiloxane
PEDOT	poly(3,4-ethylenedioxythiophene)
PEG	polyethylene glycol
PEGDA	poly(ethylene glycol) diacrylate
PEGT	poly(ethylene glycol) terephthalate
PEI	polyethyleneimine
pEtOx	poly(2-ethyl-2-oxazoline)
PEU	poly(ether urethane)
PGA	poly(glycolic acid)
PHEMA	poly(2-hydroxyethyl methacrylate)
PHIS	poly(histidine)
piBuOx	poly(2-iso-butyl-2-oxazoline)
PLA	poly(lactic acid)
PLACL	poly(L-lactide-co-ε-caprolactone)
PLGA	polylactic-co-glycolic Acid
PLLA	poly(L-lactic acid)
pMeOx	poly(2-methyl-2-oxazoline)
PMMA	poly(methyl methacrylate)
PnBA	poly(n-butyl acrylate)
PNiPAM	poly(N-isopropylacrylamide)
PPF	poly(propylene fumarate)
PPG	poly(propylene glycol)
PPM	poly(propylene maleate)
PPO	poly(propylene oxide)
pPrOzi	poly(2 N-propyl-2-oxazine)
PPy	polypyrrole
PSS	sulfonated-polystyrene
PT	polythiophene
PTMC	poly(trimethylene carbonate)
Ptriol	polycaprolactone triol
PU	polyurethane
SIBS	poly(styrene-b-isobutylene-b-styrene)
SLA	laser stereolithography
TMSPMA	3-(trimethoxysilyl)propyl methacrylate
